# Five genes as diagnostic biomarkers of dermatomyositis and their correlation with immune cell infiltration

**DOI:** 10.3389/fimmu.2023.1053099

**Published:** 2023-01-18

**Authors:** Xiaohu Zhao, Shangkun Si

**Affiliations:** College of Traditional Chinese Medicine, Shandong University of Traditional Chinese Medicine, Jinan, China

**Keywords:** bioinformatics analysis, dermatomyositis, gene expression omnibus, immune cells, machine learning

## Abstract

**Background:**

Dermatomyositis (DM) is a rare autoimmune disease characterized by severe muscle dysfunction, and the immune response of the muscles plays an important role in the development of DM. Currently, the diagnosis of DM relies on symptoms, physical examination, and biopsy techniques. Therefore, we used machine learning algorithm to screen key genes, and constructed and verified a diagnostic model composed of 5 key genes. In terms of immunity, The relationship between 5 genes and immune cell infiltration in muscle samples was analyzed. These diagnostic and immune-cell-related genes may contribute to the diagnosis and treatment of DM.

**Methods:**

GSE5370 and GSE128470 datasets were utilised from the Gene Expression Omnibus database as DM test sets. And we also used R software to merge two datasets and to analyze the results of differentially expressed genes (DEGs) and functional correlation analysis. Then, we could detect diagnostic genes adopting least absolute shrinkage and selection operator (LASSO) logistic regression and support vector machine recursive feature elimination (SVM-RFE) analyses. The validity of putative biomarkers was assessed using the GSE1551 dataset, and we confirmed the area under the receiver operating characteristic curve (AUC) values. Finally, CIBERSORT was used to evaluate immune cell infiltration in DM muscles and the correlations between disease-related biomarkers and immune cells.

**Results:**

In this study, a total of 414 DEGs were screened. *ISG15*, *TNFRSF1A*, *GUSBP11*, *SERPINB1* and *PTMA* were identified as potential DM diagnostic biomarkers(AUC > 0.85),and the expressions of 5 genes in DM group were higher than that in healthy group (*p* < 0.05). Immune cell infiltration analyses indicated that identified DM diagnostic biomarkers may be associated with M1 macrophages, activated NK cells, Tfh cells, resting NK cells and Treg cells.

**Conclusion:**

The study identified that *ISG15*, *TNFRSF1A*, *GUSBP11*, *SERPINB1* and *PTMA* as potential diagnostic biomarkers of DM and these genes were closely correlated with immune cell infiltration.This will contribute to future studies in diagnosis and treatment of DM.

## 1 Introduction

Dermatomyositis is classified as a multifactorial rare autoimmune disorder. DM is most common in children aged 4 to 14 and adults aged 40 to 60 (1). As a rare idiopathic inflammatory disease, the prevalence is estimated at 6-7 per 100,000 adults per year, with women affected twice as often as men (2). DM is characterized by characteristic specific changes, muscle weakness and it could also develop to various complications including interstitial lung disease, cardiac abnormalities, joint disorder (3). Therefore, the early diagnosis of DM was emphasized in previous clinical guidelines (4). Currently, precise diagnosis of DM is difficult because of the absence of characteristic skin lesions or myopathy (5). Myositis-Specific Antibodies, serologic antibodies exclusively associated with IIM diagnosis, have limitations. A considerable proportion of DM patients do not express MSAs and estimated diagnositic positive rates in DM range from 20% to 50% (6–9). Thus, it is crucial to find potential biomarkers that would provide the early diagnosis for DM patients.

The combination of microarray techniques and bioinformatics has facilitated the link between differentially expressed genes(DEGs) and the pathogenesis of disease. Substantial evidence, with microarray technologies, has demonstrated that IFN signatures were present in skin, muscle, and blood samples from DM patients (10). In additon, least absolute shrinkage and selection operator (LASSO) logistic regression screen significant variables *via* providing a penalty function to make the coefficients of less significant variables to 0, while support vector machine-recursive feature elimination produces the best variables through eliminating eigenvectors generated by SVM-RFE.The above methods are both of machine learning algorithms, which are already used to identify diagnostic biomarkers and offer precise models (11). Therefore, with machine learning methods screening, DEGs would be accurately identified as potential diagnostic biomarkers.

Based on current studies, immune cells play a significant role in the pathogenesis of DM. The inflammatory infiltrates of perimysial and perivascular in DM are mediated by humoral immunity and are mainly composed by CD4+ T cells and B cells (12). CIBERSORT, an immune cell infiltration algorithm, is used to analyse the content of immune cells from sample expression profiles. However, this strategy has rarely been used to explore the connection between immune cells and diagnostic biomarkers of DM.

In this study, We used the Gene Expression Omnibus database (GEO) to obtain DM-related microarray datasets and found DEGs between patients and healthy donors. We adopted machine learning techniques, namely the LASSO and SVM-RFE algorithms, to evaluate the DEGs to find potential diagnostic biomakers. we also used CIBERSORT to study the differential in immune cell infiltration in DM muscles. And by extension,we also examined the relationships between DM-related biomarkers and immune cells in order to better comprehend the immune mechanism of DM.

## 2 Materials and methods

### 2.1 Data processing and identification of DEGs

**Figure 1:**
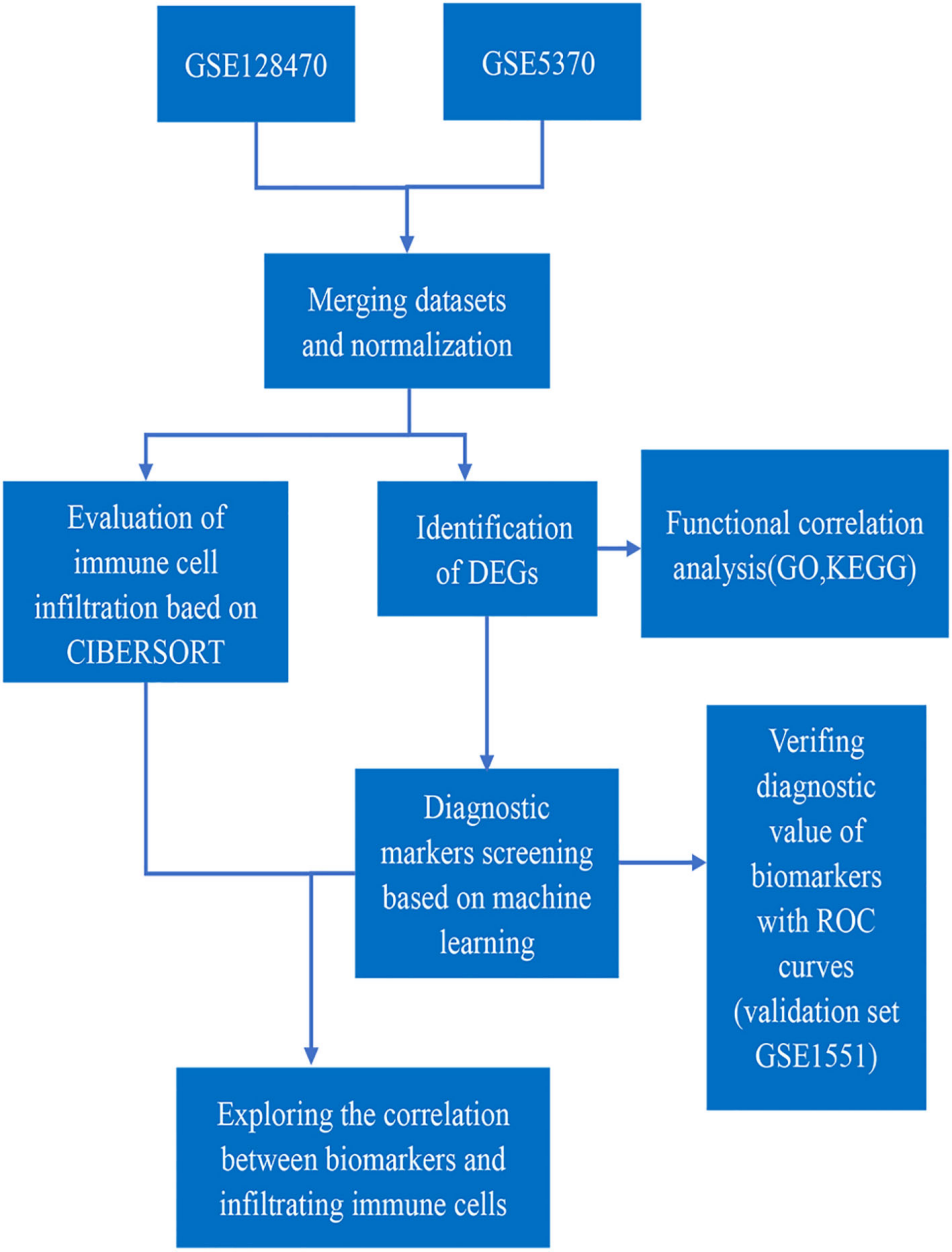
Flowchart of this study.

There Datasets GSE128470, GSE5370 and GSE1551 were downloaded from GEO database. **Table 1**. showed the sample information of the three datasets. GSE1551 was selected as the validation set, while GSE128470 and GSE5370 were merged as the test set *via* using “limma” package (16). The effect of removing inter-batch difference was visualized by PCA cluster plot. The “limma”package was used to filter DEGs between DM muscles and healthy samples with the screening criteria of *p* < 0.05 and |log_2_FC|>1. The expression difference of DEGs was visualized by the volcano map with running “ggplot2” package (17). The overall process of this study is shown in **Figure 1**.

**Table 1:** Basic information on DM muscle datasets.

Datasets (Platform)	Country	Organism	Number of samples (DM/NC)	Gender (M/F)	Age	Treated/not	Diagnostic criteria	Reference
GSE5370 (GPL96)	USA	Human skeletal muscle	5/4	0/5	Adult	0/5	Not described	Citation missing
GSE128470 (GPL96)	USA	Human skeletal muscle	12/12	0/15	Adult	Not described	Hoogendijk et al. (13)	Greenberg et al. (14)
GSE1551 (GPL96)	USA	Human skeletal muscle	14/10	10/4	Adult	7/7	Hoogendijk et al. (13)	Greenberget al. (15)

### 2.2 Functional enrichment analyses of DEGs

We used the “clusterProfiler” package (18) to analyse KEGG pathway and GO enrichment analyses of DEGs. The results with *p.adjust value* < 0.05 (Benjamini-Hochberg) were considered as statistically significant and were visualized by using “ggplot2” package.

### 2.3 Machine learning-based biomakers screening

The LASSO algorithm and SVM-RFE algorithm were used to select characteristic features to screen DM diagnostic biomakers, and we used “glmnet” (19) and “caret” packages to perform machine learning algorithms. These genes overlapping between LASSO and SVM-RFE algorithms were extracted and used for subsequent analyses.

### 2.4 CIBERSORT-based immune cell infiltration analysis

CIBERSORT was used to analyze 22 types of different immune cell infiltration in DM muscles with the condition of *p* < 0.05. The correlation of immune cells was visualized by a heatmap with running “corrplot” package (20), and “ggpubr” package was used to draw a box plot to show the difference of immune cells between DM muscles and healthy samples. The GSE1551 dataset was used to verify the difference in expression of diagnostic genes between the DM group and the healthy group and the results were visualized by using “ggplot2” package.

### 2.5 Correlation analysis between immune cell infiltration and diagnostic biomarkers

Correlations between diagnostic biomakers and immune cells infiltration were evaluated *via* spearman correlation analysis and the results were visualized by using “ggpubr” package.

## 3 Results

### 3.1 Data processing and DEGs identification

**Figure 2:**
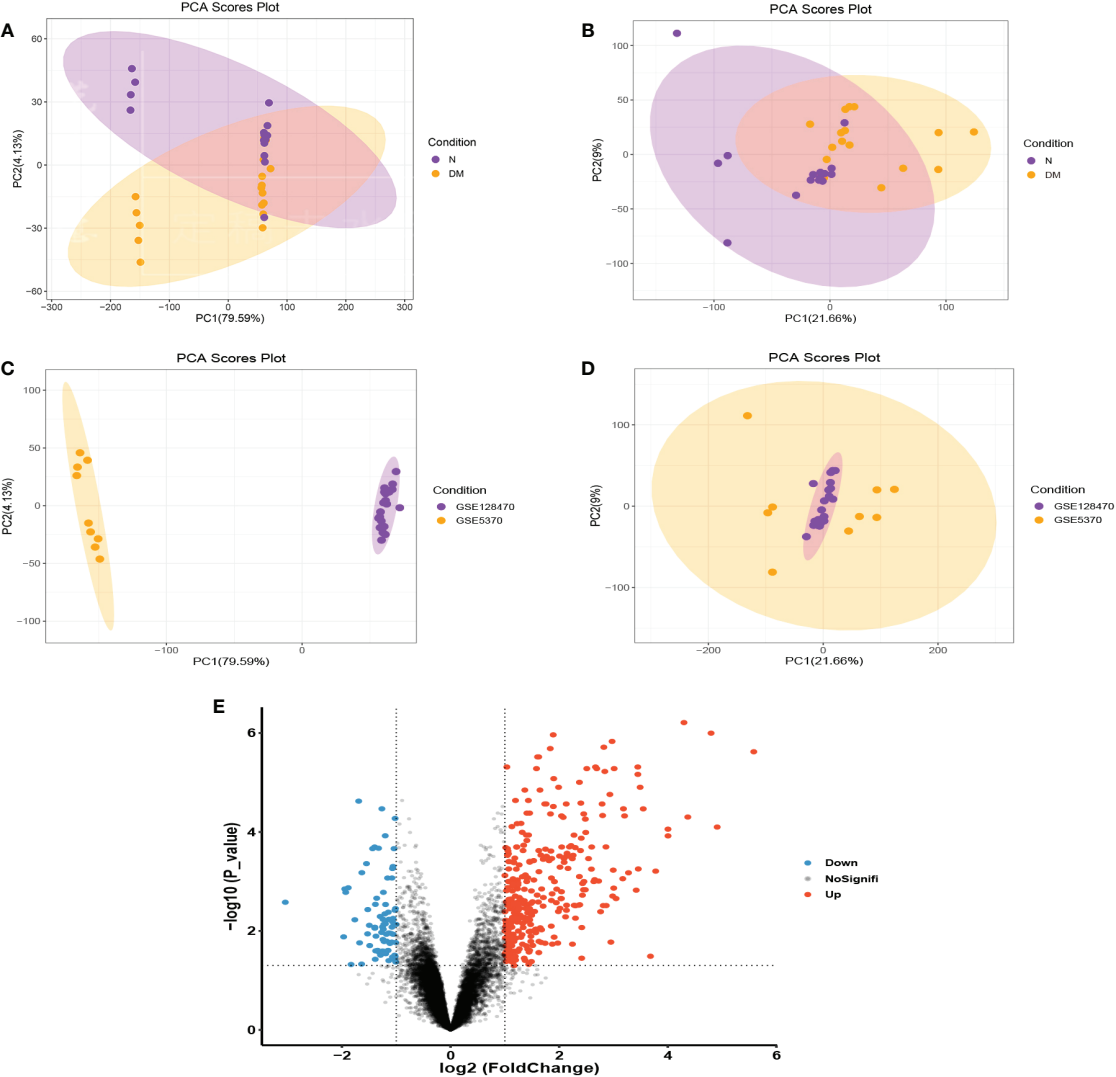
**(A, B)** Before and after normalization, two-dimensional PCA cluster diagrams of the GSE128470 and GSE5370 datasets. **(C, D)** Before and after normalization, two-dimensional PCA cluster diagrams of normal and DM samples. **(E)** In the combined dataset, a volcano map shows differentially expressed genes.

GSE128470 and GSE5370 were combined and the two-dimensional PCA cluster diagrams (**Figures 2A–D**) were used to present the effect of eliminating the inter-batch difference of the merged dataset. Then a total of 414 DEGs were obtained from the merged gene expression matrix, and the results were shown in the volcano plot (**Figure 2E**).

### 3.2 Functional enrichment analysis of DEGs

**Figure 3:**
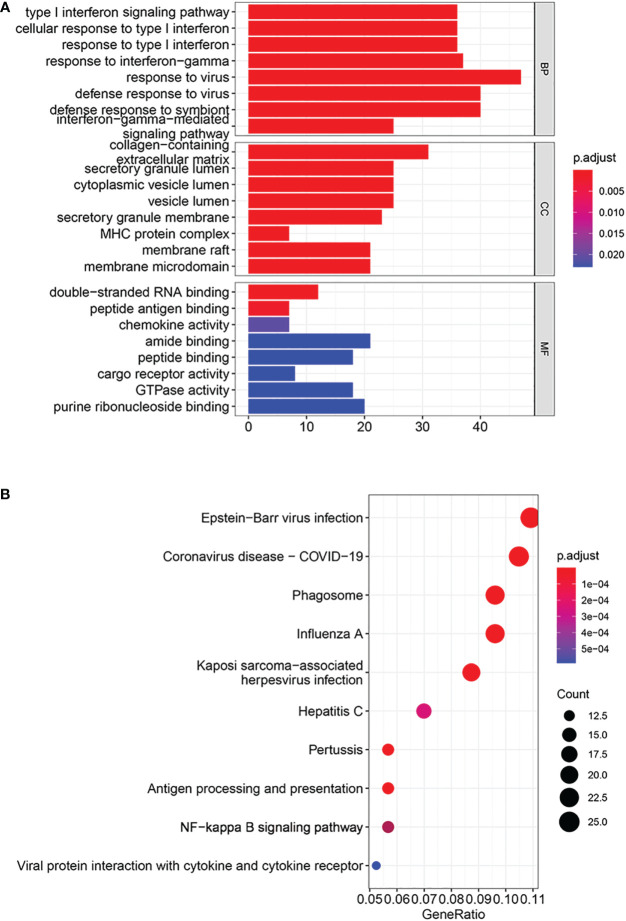
Analyses of functional enrichment. **(A)** GO enrichment analysis by DEGs. **(B)** KEGG pathways enriched by DEGs.

Based on GO enrichment analysis, with respect to biological process, DEGs were primarily enriched in the type I interferon signaling pathway, response to interferon-gamma, and response to virus. With respect to cellular components, DEGs were mainly enriched in collagen-containing extracellular matrix, secretory granule lumen and MHC protein complex. In terms of Molecular function, DEGs were significantly related to double-strand RNA binding, amide binding and purine ribonucleoside binding (**Figure 3A**). KEGG analysis results showed that DEGs were primarily enriched in pathways about innate immunity and response to virus (**Figure 3B**).

### 3.3 Diagnostic biomarkers identification and validation

**Figure 4:**
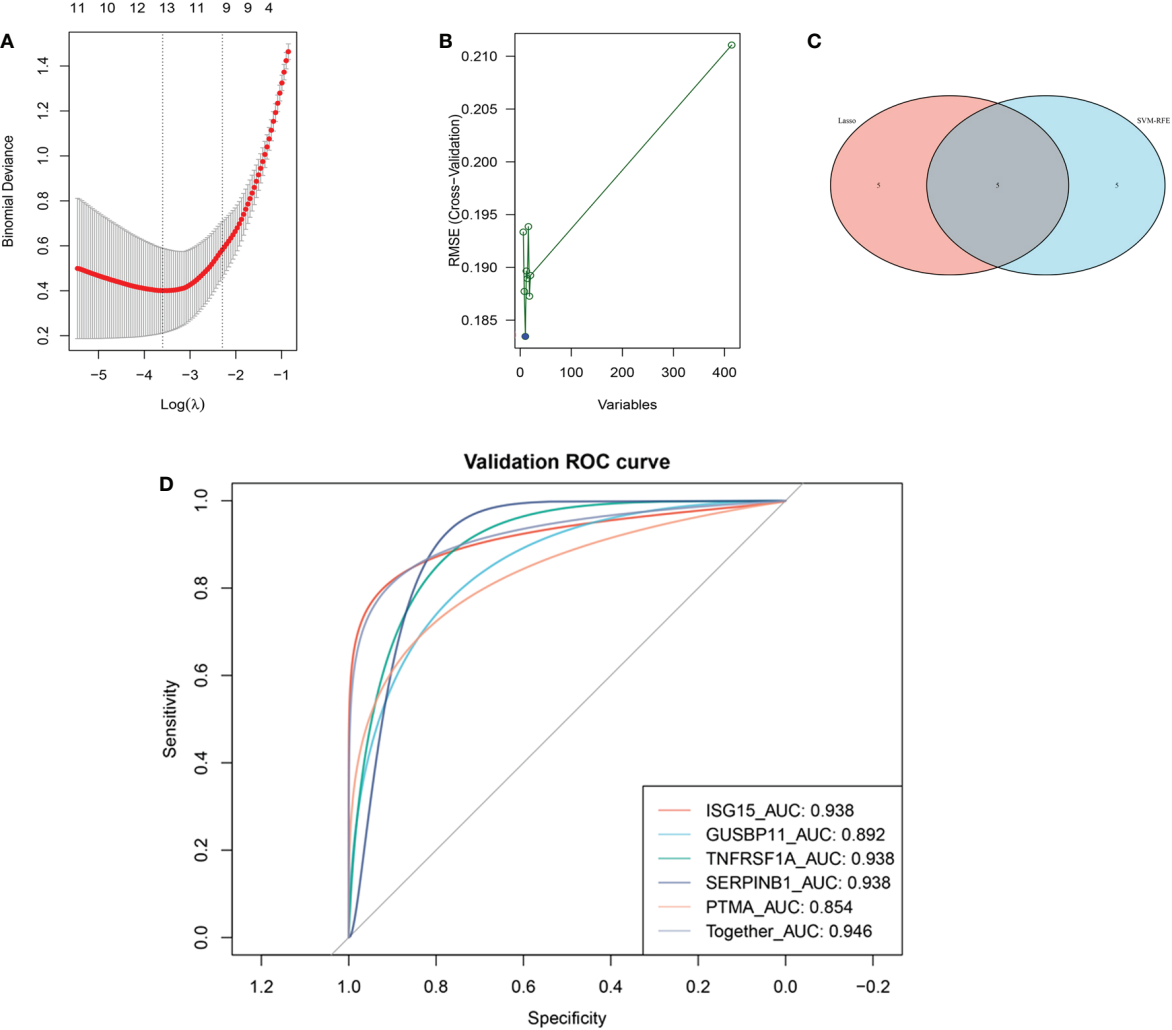
Machine learning-based screening biomarkers and verification with ROC curves. **(A)** Ten genes screened by LASSO logistic regression algorithm. **(B)** Ten genes screened by SVM-RFE algorithm. **(C)** The five overlapping genes obtained by both the LASSO and SVM-RFE algorithms. **(D)** ROC curves for the diagnostic biomarkers in the validation dataset GSE1551.

**Figure 5:**
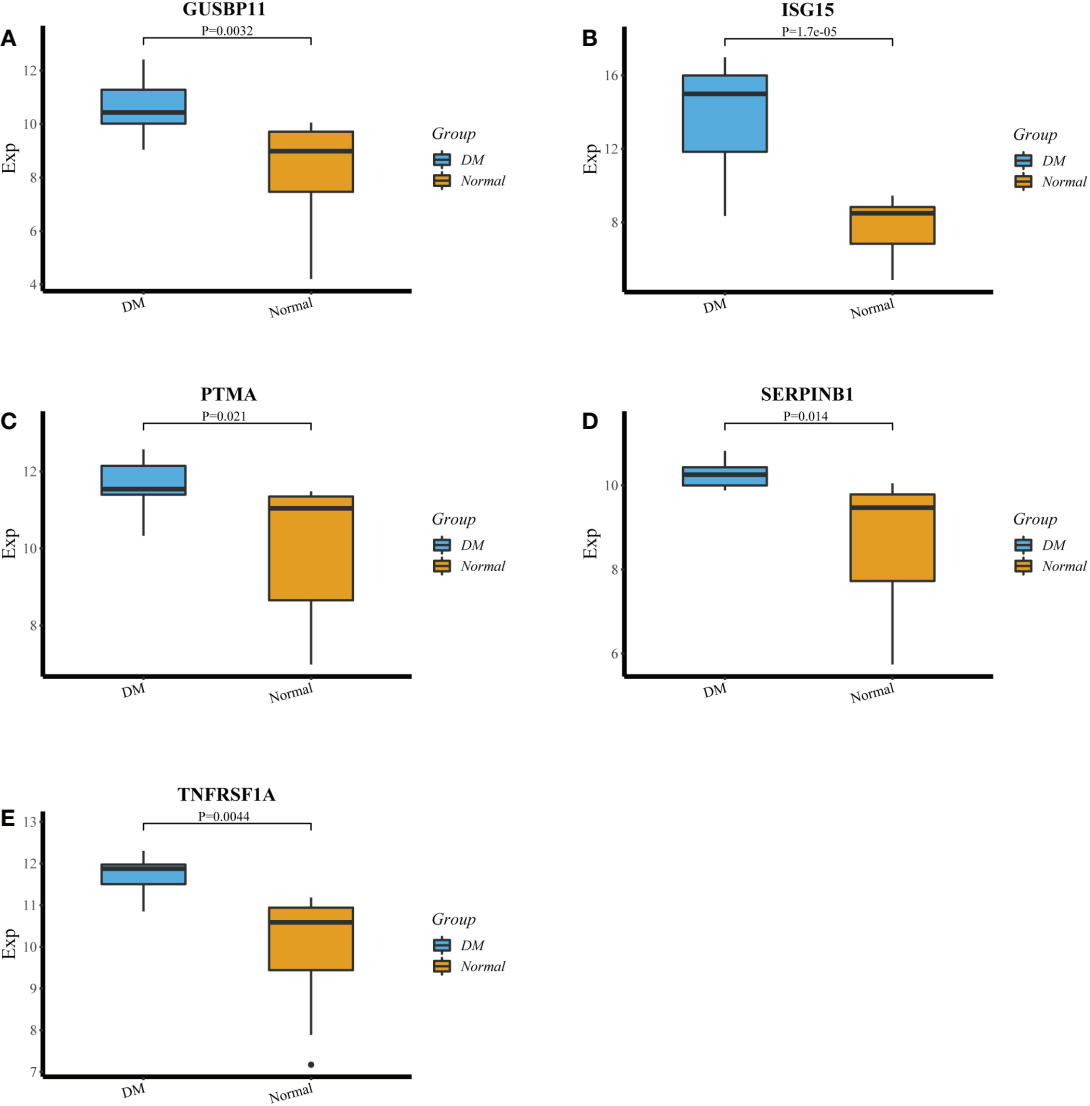
**(A‐E)** The expression of *GUSBP11,ISG15*, *PTMA*, *SERPINB1* and *TNFRSF1A* in DM group and healthy group.

The LASSO algorithm identified 10 genes as potential DM diagnostic biomarkers from 414 DEGs (**Figure 4A**), and SVM-RFE algorithm also identified 10 genes (**Figure 4B**). 5 genes selected by two machine learning algorithms were overlapped (**Figure 4C**), including *ISG15*, *TNFRSF1A*, *GUSBP11*, *SERPINB1* and *PTMA*. They were fitted into one variable, and its diagnostic efficiency presented a extremely high level (AUC = 0.946) in validation set (GSE1551). In addition, 5 genes respectively had high diagnostic value (AUC > 0.85, **Figure 4D**), demonstrating that the diagnostic model of *ISG15*, *GUSBP11*, *TNFRSF1A*, *SERPINB1* and *PTMA* had reliable diagnostic value in DM. The expressions of 5 genes in DM group were higher than that in healthy group(*p* < 0.05, **Figures 5A–E**).

### 3.4 Evaluation and correlations analysis of immune cell infiltration

**Figure 6:**
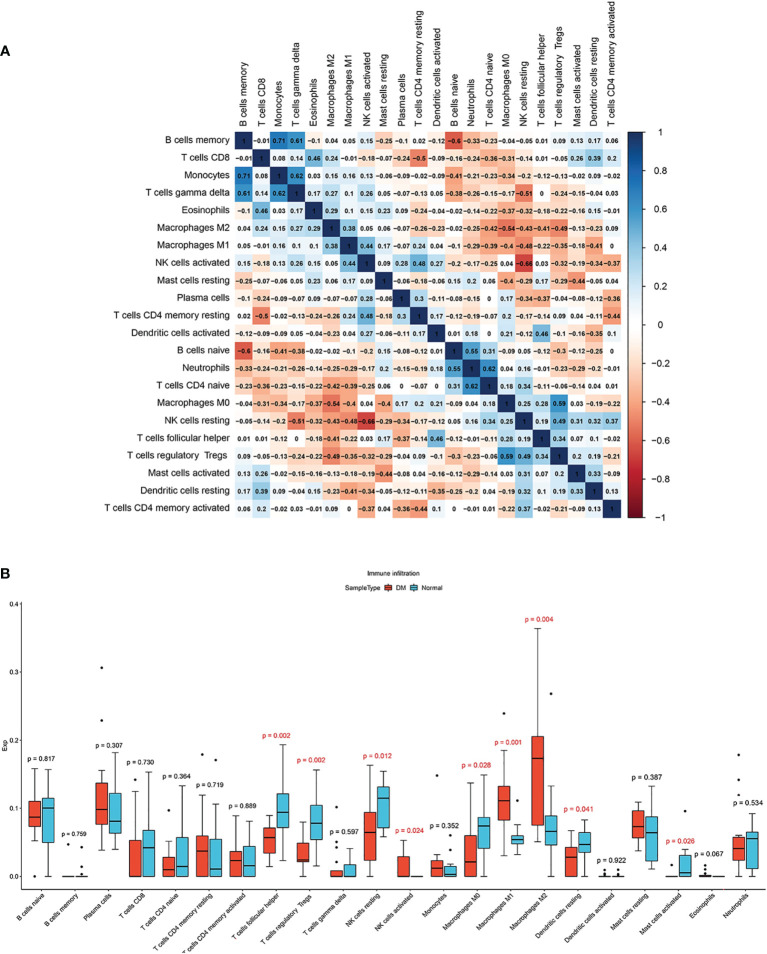
**(A)** Correlation heatmap among 22 types of infiltrating immune cells. **(B)** Box plot of differences of 22 immune infiltrating cells. Normal and DM groups are represented by blue and red, respectively.

With correlations heatmap demonstrating (**Figure 6A**), B memory cells and Monocytes cells had strong positive correlations(*r* = 0.71). activated NK cells and resting NK cells had significant negative correlations(*r* = -0.66). The differences of immune cell infiltration were shown by the box plot. Compared to healthy samples, activated NK cells, M1 and M2 Macrophages(*p* < 0.05) infiltrated more in DM muscles, while T follicular helper cells, regulatory T cells, resting NK cells, M0 Macrophages, and resting Dendritic cells (*p* < 0.05) infiltrated less in DM muscles (**Figure 6B**).

**Figure 7:**
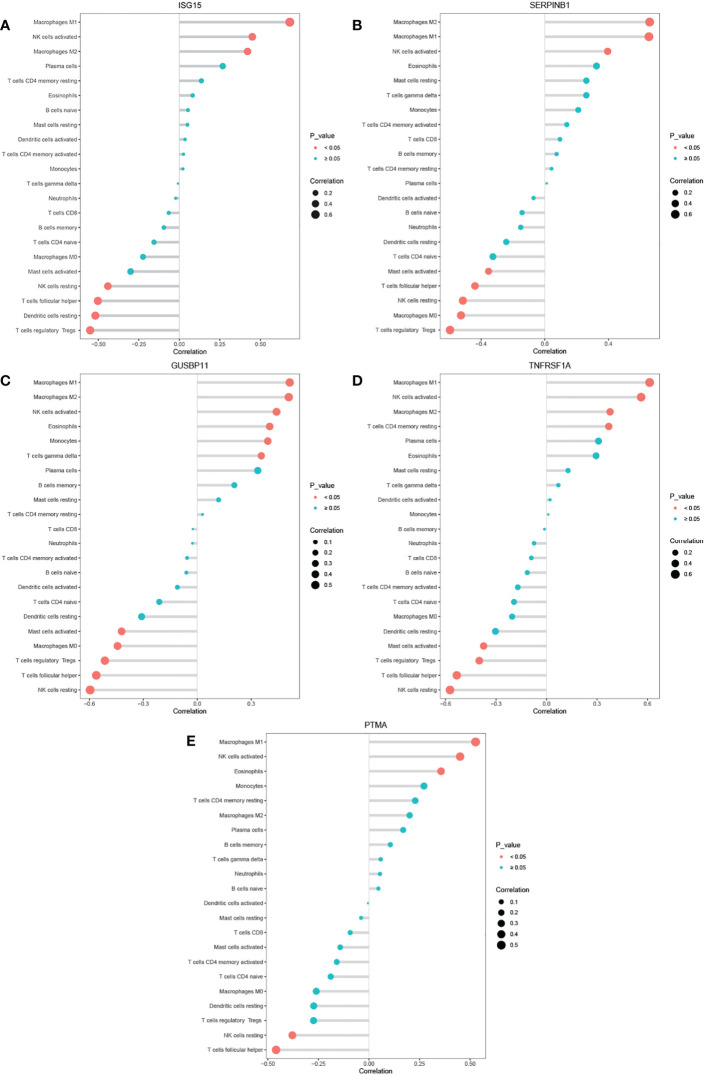
**(A–E)** Correlation between *ISG15*, *SERPINB1*,*GUSBP11*, *TNFRSF1A*, *PTMA* and infiltrating immune cells.

The threshold of significant positive correlation was *r* > 0.4 and *p* < 0.05, while that of negative correlation was *r* < -0.4 and *p* < 0.05. Correlations analysis found that the expression of *ISG15* positively correlated with activated NK cells, M1 and M2 Macrophages, and negatively correlated with T follicular helper cells, regulatory T cells, resting NK cells and resting dendritic cells (**Figure 7A**); *SERPINB1* positively correlated with M1 and M2 Macrophages and negatively correlated with T follicular helper cells, regulatory T cells, resting NK cells and M0 Macrophages (**Figure 7B**); *GUSBP11* negatively correlated with T follicular helper cells, regulatory T cells, resting NK cells and M0 Macrophages, while positively correlated with M1 and M2 Macrophages and Eosinophils cells (**Figure 7C**); *TNFRSF1A* positively correlated with M1 Macrophages and activated NK cells, and negatively correlated with T follicular helper cells, regulatory T cells and resting NK cells (**Figure 7D**); *PTMA* positively correlated with M1 Macrophages and activated NK cells, and negatively correlated with T follicular helper cells (**Figure 7E**).

## 4 Discussion

Dermatomyositis is an autoimmune disease that causes muscle weakness, skin lesion and multiple organs complications. Present studies show that IFN-1 inducible immune response play important roles in the pathogenesis of DM (21, 22). Bioinformatics technology and microarray expression have been utilized for exploring diagnostic markers. However, there was less research analyzing the relationship between diagnostic markers and infiltrating immune cells in DM muscle samples. Our research applies machine learning algorithms and ROC curves to screen biomarkers with great diagnostic efficacy, as well as the CIBERSORT algorithm to explore associations between immune cell infiltration and these biomarkers.

### 4.1 The importance of immune responses in DM

We found 414 DEGs after downloading and merging two DM-related datasets from the GEO database. According to function enrichment analyses, response to viral infection and innate immunity has essential roles in the pathogenesis of DM. A number of studies has proved that virus infection is linked to DM, such as COVID-19 virus (23). As for the enrichment of immune-related functions, significant IFN-1 signature has been found in the muscle, blood, and skin in DM patients (21). The production of IFN-1 is mainly mediated by toll-like receptor (TLR) signaling pathway (24). TLR4 in the TLR pathway is a pivotal receptor in the immune system in the pathogenesis of DM. By activating the MyD88 signaling pathway and its downstream NF-κB pathway, TLR4 ultimately upregulates the strong pro-inflammatory factor IFN-γ and forms a pro-inflammatory myopathy environment (25). Our enrichment results are consistent with the above studies and we further stress the importance of immune response and inflammatory in DM muscles.

### 4.2 The value of the identified diagnostic genes for DM

We identified and validated *ISG15*, *GUSBP11*, *TNFRSF1A*, *SERPINB1* and *PTMA* as potential diagnostic biomarkers using mechine learing algorithms and ROC curves. Interferon-stimulated gene 15(*ISG15*), a kind of ubiquitin-like protein, is induced by Interferon-α/β(IFN-α/β) and acts as a key negative regulator in type 1 IFN pathway for autoinflammatory response caused by overreaction of IFN-α/β signaling pathway (26). Among inflammatory mypathies, the upregulation of *ISG15* transcript is unique to DM, which is one of the most strongly up-regulated genes (27). The IFN-1 score composed of thirteen genes can be utilized to reliably assess type 1 IFN pathway activation in DM, while *ISG15* expression level alone could be used to assess it perfectly, showing potential diagnostic ability of *ISG15* in DM (28). In this study, *ISG15* is regarded as a good diagnostic biomarker, which is consistent with previous studies. Tumor Necrosis Factor Receptor-1(*TNFRSF1A*, also known as *TNFR1*), as a transmembrane glycoproteins with 55kDa molecular, is characterized by death domain on intracellular region (29). Studies have indicated that TNF-TNFR1 signaling mainly shows effects on pro-inflammatory and cell death, which related to many diseases including autoimmune syndromes, COVID-19, and cancer (30, 31). Thus, selective inhibitors for *TNFR1* are considered as next-generation anti-TNF treatment in autoimmune diseases (32). Moreover, the serum level of s*TNFR1* in active DM patients is significantly higher than patients with inactive DM, which suggested *TNFR1* is related to activation of inflammatory during acute phase of DM (33). The above research has proved that *TNFRSF1A* has great significance in both disease diagnosis and treatment.

But there is no research about the role of *GUSBP11*, *PTMA* and *SERPINB1* in DM. Glucuronidase, b pseudogene 11(*GUSBP11*), a long non-coding RNA, has been proved that might affect the development of tumor in recent research, but its molecular mechanism has not been explored clearly enough (34–36). Prothymosin-α(*PTMA*) belongs to the α-thymosin family (37). As a kind of acidic nuclear protein with localization signal, *PTMA* plays an important role in various aspects including immunological functions, cell proliferation and apoptosis (38). The expression of *PTMA* has been used to predict poor prognosis in several tumor diseases (39, 40). Serine protease inhibitor, clade B, member1(*SERPINB1*, also known as *LEI*), a member of the *SERPINB* family of proteins, protects cells from their own proteases during stress. *SERPINB1* shows two special enzyme activities, one is antiprotease activity relaying on its site loop and the other is endonuclease activity dependent on the cleavage of the reactive site loop (41). In a *SERPINB1*-deficient mice model study, *SERPINB1* plays an important role in protecting lung antimicro proteins from proteases during infection (42). Moreover, *SERPINB1* maintains the survival of neutrophils and also is related to IL-17-expressing T cells (43). In our study, *GUSBP11*, *PTMA* and *SERPINB1* show high expression and great diagnostic efficacy in DM(AUC = 0.892, 0.854 and 0.938). However, the role and molecular regulatory mechanism of these genes in DM remain unclear, which suggests that its potential therapeutic value requests further studies.

### 4.3 Associated immune cell infiltration in DM muscles

We used the CIBERSORT algorithm to explore the difference of 22 type of immune cell infiltration between DM muscles and normal samples, and conducted the association analysis between 5 genes with diagnostic value and CIBERSORT results for the first time. To our surprise, *ISG15*, *GUSBP11*, *TNFRSF1A*, *SERPINB1* and *PTMA* all significantly positively correlated with M1 macrophages, while significantly negatively correlated with Tfh cells. In addition, 4 genes of them (except *PTMA*) positively correlated with Actived NK cells, while negatively correlated with Resting NK cells and Treg cells. The above immune cells significantly correlated with diagnostic genes and also showed significant differences in CIBERSORT results. Therefore, further study on the up-regulation of M1 macrophages, activated NK cells, and down-regulation of Tfh cells, Resting NK cells and Treg cells in DM will be of value for the diagnosis and treatment of DM.

In previous studies, M1 macrophages, a type of macrophage activated by specific factors, can produce pro-inflammatory cytokines and easily lead to tissue damage (44). Shogo Matsuda et al. (45) who studied the role of M1 macrophages in the pathology of DM-ILD, speculated that activated monocytes and Th1 cells promoted the expression of IL-2 and CXCL11, inducing the differentiation of M1 macrophages, and then M1 macrophages promoted the expression of IL-8 and IL-18 to activate neutrophils and produced pro-inflammatory cytokines such as IL-6 and TNF-α. T follicular helper (Tfh) cells, a subset of CD4+ T cells, play an important role in orchestrating B cells to maintain humoral immune response (46). Xiaoyu Hou et al. have used flow cytometry to found that the proportion of circulating Tfh cells declined in DM patients compared with that of healthy samples (47). However, previous studies found that overexpanded Tfh cells, especially peripheral Tfh cells, were observed in other autoimmune diseases (48), which suggests that the special role of Tfh cells in DM deserves further studies. Treg cells, a subset of CD4 + T cells, play a significant role in the anti-inflammatory effects of skeletal muscle and skin, and the Th17/Treg imbalance is linked to the incidence and progression of DM (49–51). The levels of peripheral Treg cells in DM patients are also lower than that in normal persons, possibly due to the regulation of RUNX3-Foxp3 by HAGLR (52, 53). Nature killer (NK) cells, a subset of innate lymphoid cells, play an important role in inflammation and autoimmune responses *via* cell migration, cytotoxicity, and cytokine production (54). However, the specific mechanism of NK cells remains unclear in IIMs (54). According to our CIBERSORT results, there were only resting NK cells in normal muscle tissue. But in DM muscle, the resting NK cells significantly decreased and the activated NK cells significantly increased. In previous studies, the levels of periphery NK cells in DM patients were decreased, which may be related to NK cells migration into muscle (54, 55). Therefore, we speculated that the up-regulation of activating NK cells infiltration in DM muscle was related to the migration of peripheral cells and the activation of resting NK cells in muscle.

### 4.4 Limitations

There are some limitations to our study. For example, although the diagnostic model has clinical significance, it needs to recruit patients to provide muscle samples for further verification. Only the correlations between immune cell infiltration and diagnostic genes was proposed, and the mechanism of these genes affecting immune cells still needs to be explored by designing experiments.

## 5 Conclusion

In conclusion, we identified *ISG15*, *TNFRSF1A*, *GUSBP11*, *SERPINB1* and *PTMA* as potential diagnostic biomarkers of DM and we also found that these genes were significantly related to M1 macrophages, activated NK cells, Tfh cells, resting NK cells and Treg cells. Further exploring these immune-related biomarkers and immune cells will be of value to confirm mechanism of immune cell infiltration in DM.

## Data availability statement

The original contributions presented in the study are included in the article/**Supplementary Material**. Further inquiries can be directed to the corresponding author.

## Author contributions

XZ confirmed the initial idea, wrote the manuscript and operated the software. SS was responsible for data collection. XZ reviewed and edited the manuscript. All authors contributed to the article and approved the submitted version.

## Supplementary material

Supplementary Table 2 Information about the sample on the chip(GSE128470).

Supplementary Table 3 Information about the sample on the chip(GSE5370).
