# Supplementary material for: Five genes as diagnostic biomarkers of dermatomyositis and their correlation with immune cell infiltration
Source: Front Immunol. 2023 Jan 18;14:1053099. doi: 10.3389/fimmu.2023.1053099 (PMC9889851; doi:10.3389/fimmu.2023.1053099)
Supplement: Supplementary file 1 [file Table_1.docx]

Supplementary Material

Five Genes as Diagnostic Biomarkers of Dermatomyositis and Their Correlation with Immune cell Infiltration

Xiaohu ZHAO, Shangkun SI*

*** Correspondence:** Corresponding Author: [359833468@qq.com](mailto:359833468@qq.com)

1. Supplementary Tables

**Supplementary Table S1. Full name of the abbreviation**

| Abbreviations | Full name |
| --- | --- |
| AUC | Area under the receiver operating characteristic curve |
| BP | Biological process |
| CC | Cellular component |
| CXCL11 | C-X-C Motif Chemokine Ligand 10 |
| DEGs | Diferentially expressed genes |
| DM | Dermatomyositis |
| DM-ILD | Dermatomyositis interstitial lung disease |
| FOXP3 | Forkhead Box P3 |
| GEO | Gene Expression Omnibus |
| GO | Gene Ontology |
| *GUSBP11* | Glucuronidase, b pseudogene 11 |
| HAGLR | HOXD Antisense Growth-Associated Long Non-Coding RNA |
| IFN-1 | Type 1 interferon |
| IFN-γ | Interferon-gamma |
| IIMs | Idiopathic inflammatory myopathies |
| *ISG15* | Interferon-stimulated gene 15 |
| KEGG | Kyoto Encyclopedia of genes and genomes |
| LASSO | Least absolute shrinkage and selection operator |
| MF | Molecular function |
| MSAs | Myositis-Specific Antibodies |
| NK cells | Nature killer cells |
| *PTMA* | Prothymosin-α |
| RUNX3 | RUNX Family Transcription Factor 3 |
| *SERPINB1* | Serine protease inhibitor, clade B, member1 |
| SVM-RFE | Support vector machine recursive feature elimination |
| Tfh cells | T follicular helper cells |
| TLR4 | Toll-like receptor 4 |
| TNF | Tumor necrosis factor |
| *TNFRSF1A* | Tumor Necrosis Factor Receptor-1 |
